# Psychosocial distress and associated factors among adult cancer patients at oncology: a case of Ethiopia

**DOI:** 10.3389/fonc.2023.1238002

**Published:** 2023-12-21

**Authors:** Astewle Andargie Baye, Sitotaw Kerie Bogale, Abebu Tegenaw Delie, Mengistu Melak Fekadie, Haileyesus Gedamu Wondyifraw, Mengistu Ewunetu Tigabu, Mulu Kebede

**Affiliations:** ^1^ Department of Adult Health Nursing, College of Health Sciences, Debre Tabor University, Debre Tabor, Ethiopia; ^2^ Department of Adult Health Nursing, School of Health Sciences, Bahir Dar University, Bahir Dar, Ethiopia; ^3^ Department of Pediatric and Child Health Nursing, College of Health Sciences, Debre Tabor University, Debre Tabor, Ethiopia

**Keywords:** Amhara region, cancer, Ethiopia, factors, prevalence, psychosocial distress

## Abstract

**Background:**

Psychosocial distress is a chronic burden for cancer survivors, which impacts both their quality of life and their oncologic prognosis. Although the national cancer prevention and control program in Ethiopia has made efforts in cancer prevention, control, and management by implementing the national cancer control plan 2016–2020, there was no enough evidence about psychosocial distress among adult cancer patients. So, it is critical to understand the magnitude of psychosocial distress and the factors that contribute to it.

**Objective:**

The purpose of this study was to assess the prevalence of psychosocial distress and associated factors among adult cancer patients at oncology units in the Amhara regional state, Ethiopia. 2022.

**Methods:**

A multicenter institution-based cross-sectional study was conducted among a sample of 605 adult cancer patients from 30 April to 22 June 2022. A systematic random sampling technique was employed to select the study units. In addition, data were collected through interviewers administered questionnaires by using the validated and pretested tools. Distress was assessed using the Questionnaire on Stress in Cancer Patients Revised 10. Both bivariable and multivariable logistic regression was used to describe the association between dependent and independent variables. Independent variable with *p* < 0.25 in the bivariable logistic regression analyses were entered into multivariable logistic regression model. Variables with *p* < 0.05 in the multivariable logistic regression analyses were considered as statistically significant associated factors of psychosocial distress.

**Result:**

A total of 593 adult cancer patients took part in this study with mean age of 46.86 ± 14.5 years. The overall prevalence of psychosocial distress was 63.74%. Variables such as being female [AOR = 1.98, 95% confidence interval (CI): 1.24–3.17], patients who lives in rural areas (AOR = 2.3, 95% CI: 1.49–3.54), community-based health insurance utilization (AOR = 0.34, 95% CI: 0.23–0.51), patients on chemotherapy treatment (AOR = 2.72, 95% CI: 1.38–5.39), patients with comorbidity (AOR = 3.2, 95% CI: 1.67–6.10), and symptom burdens such as severe fatigue (AOR = 1.65, 95% CI:1.09–2.39) and severe nausea (AOR = 2.07, 95% CI: 1.43–3.00) were statistically associated with psychosocial distress.

**Conclusion and recommendation:**

In general, the findings of this study showed a relatively high magnitude in which around two-thirds of patients experienced psychosocial distress. It is better to establish and enforce the integration and coordination of psychosocial oncology service programs at national level with parallel guidelines and policies.

## Background

Cancer is a rapidly increasing global burden, surpassing current control capacity in sub-Saharan Africa, including Ethiopia (1, 2). It is commonly perceived as a life-threatening and potentially traumatic illness (3). Patients who received cancer diagnosis and undergo its treatment experience a variety of dreadful issues and comorbidities that involve medical, physical, and emotional aspects (4, 5). One of the most prevalent comorbidity is cancer-related psychosocial distress (6). The National Comprehensive Cancer Network (NCCN) defined distress in cancer as “a multifactorial unpleasant experience of a psychological (i.e., cognitive, behavioral, emotional), social, spiritual, and/or physical nature that may interfere with one’s ability to cope effectively with cancer, its physical symptoms, and its treatment (7).” Psychosocial distress is a chronic burden for cancer survivors, which impacts both their quality of life and their oncologic prognosis (8). The magnitude of psychosocial distress in the cancer survivorship cohort is reported as between 20% and 52% (9). About 50% of cancer patients have clinically significant unrecognized or untreated distress during the cancer trajectory (10). It varies between economically developed and underdeveloped countries, with developing countries having the greatest magnitude (11). A study conducted across 55 North American cancer centers found that 46% of cancer patients suffer from psychosocial distress (12). A multicenter study conducted in Italy found that 26.6% of cancer patients were psychosocially distressed (13). Another study in tertiary care institutes in Saudi Arabia and Sri Lanka revealed that the overall prevalence of psychosocial distress was 46% and 65%, respectively (14, 15). On the other hand, high magnitude of psychosocial distress was found according to studies conducted in Cameroon and Kenya, in which about 69.2% and 72.2% of cancer patients were distressed (16, 17). A study conducted in Ethiopia reported that the magnitude of anxiety and depression was 64.9% and 47.4%, respectively (18).

Regarding factors of distress, previous studies have revealed that estimates of the magnitude of distress vary based on the type and stage of cancer (19). High magnitudes of clinically significant level of distress were found among patients with hematologic, lung, and head and neck cancers (10, 20). Advanced stages of cancer, treatment options, and number of uncontrolled symptom burdens such as fatigue, pain, anxiety, difficulty in transportation, changes in role relationships, physical limitation, and fear of recurrence also contribute to distress (21, 22). Psychosocial distress has also been observed to vary by patient demographics, in which female gender (23) and older age were associated with higher distress (24). Moderate to severe levels of distress have resulted from being rural residents, low educational status, and divorced patients (25). Evidence from the NCCN reveals that inadequate social support, living alone, severe co-morbid illnesses, financial problems, and spiritual/religious concerns put cancer patients at an increased risk of psychosocial distress (26).

Distress in patients with cancer is associated with various negative outcomes, such as reduced adherence to treatment (27), increased treatment toxicities (28), trouble-making decisions about treatment (9), increased morbidity (6), poor quality of life, and lower chance of surviving (29). It is also associated with increased cancer-specific premature mortality, even at lower levels of psychosocial distress (30, 31). Long-term distress can lead to significant financial toxicity, with cancer survivors experiencing higher annual medical expenses compared to those without distress history (32, 33).

Despite the fact that distress is significant among cancer patients, it frequently remains under-recognized and under-treated, and interventions that can be helpful are not always delivered (34). Potential interventions targeting exercise intensity, character strengths, medical coping, pharmacologic interventions, social work and counseling services, psychotherapy, cognitive behavioral therapy, and spiritual care are all important aspects used to reduce the burden of psychosocial distress (9, 35). According to International Psycho-Oncology Society; to bring high quality, cancer care must comprise psychosocial domain in regular basis and distress management should be recognized as universal human right (36). The NCCN also suggested psychosocial distress as the sixth vital sign, recommending its assessment after checking temperature, pulse, respiration, blood pressure, and pain (37, 38). As a result, various countries throughout the world are implementing psychosocial distress screening programs by incorporating psycho-oncology services (39, 40). However, the recognition of distress and the implementation of psychosocial oncology program are fragmented and undeveloped, particularly in African countries (41). Implementing psycho-oncology programs in cancer treatment could improve the prognosis of cancer patients (42). Although the national cancer prevention and control program in Ethiopia has made efforts in cancer prevention, control, and management by implementing the national cancer control plan 2016–2020 in order to achieve the long-term goal of reducing cancer morbidity and mortality through early detection and screening, diagnosis, and provision of comprehensive intervention (43), no enough evidence about psychosocial distress among cancer patients in our sample. So, it is critical to understand the magnitude of psychosocial distress and the factors that contribute to it in order to screen and treat it as soon as possible. The findings of this study could serve as an evidence-based resource for decision and policymakers in establishing and enforcing the integration of psycho-oncology services. It could also create opportunities for the implementation of psychosocial services.

## Materials and methods

### Study design

Multicenter, institution-based cross-sectional study was conducted to identify the prevalence of psychosocial distress and associated factors among adult cancer patients attending at oncology units in the Amhara regional state, Ethiopia.

### Study area and period

The study was conducted at public health facilities with oncology units in the Amhara regional state from 30 April to 22 June 2022 among cancer patients who were attending for treatment and follow-up. Currently, the Amhara regional state has four specialized referral hospitals that provide comprehensive and integrated cancer care. The four institutions are Felege Hiwot Comprehensive Specialized Hospital (FHCSH), University of Gondar Comprehensive Specialized Hospital (UOGCSH), Dessie Referral Hospital (DRH), and Tibebe Ghion Specialized Hospital (TGSH). Both FHCSH and TGSH were found in Bahir Dar City, the capital of Amhara regional state, which is 565 km apart from Addis Ababa, Ethiopia. UOGCSH and DRH, on the other hand, are 738 and 396.8 km from Ethiopia’s capital, respectively. These oncological care facilities have been operating since 2015, with the help of a few devoted individuals, and provide comprehensive oncology care for a variety of cancer patients. Currently, region’s oncology units, such as FHCSH, UOGCSH, DRH, and TGSH have 22, 32, 16, and eight inpatient beds for the treatment of cancer patients, respectively.

### Source population

All adult cancer patients attending at oncology units in the Amhara regional state.

### Study population

All adult cancer patients attending at oncology units in the Amhara Regional state during the study period (30 April to 22 June 2022).

### Study unit

Each adult cancer patient at oncology units in the Amhara regional state that fulfilled the inclusion criteria participated in this study.

### Eligibility criteria

#### Inclusion criteria

All cancer patients whose age ≥ 18 years and who were attending at each oncology units were included into the study.

#### Exclusion criteria

Critically sick adult cancer patients who were unable to give response and had no attendants during the data collection period.

### Sample size determination and sampling procedure

First, oncology units, which are located in the Amhara regional state, were identified. According to the unit’s cancer registry of patients with cancer, each hospital, such as FHCSH, UoGCSH, DRH, and TGSH, treated about 420, 392, 224, and 70 cancer patients on average every month, respectively. Moreover, averagely, the overall number of cancer patients who have been followed up on each month at the region’s oncology units was 1,106. The sample was determined by using a single population proportion formula by considering the following statistical assumptions: P = proportion of patients with cancer, who experience psychosocial distress (50%), Z/2 = 95% CI Z score, d = margin of error (5%). The final sample size was 605, considering 1.5 design effect and 10% non-respondent rate. An independent and representative sample was determined for each oncology unit by using a stratified sampling technique based on the above information. Also, the final sample size was determined after being proportionally assigned to each oncology unit, with 230 from FHCSH, 214 from UOGCSH, 123 from DRH, and 38 from TGSH. A systematic random sampling technique was employed to select the final actual participant samples. The *k* ^th^ value in the systematic random sampling procedure was calculated by dividing the source population by the total sample size. Based on the monthly attending adult cancer patients at oncology units the *k* ^th^ value became 2. Finally, the actual study participants were selected with every second cancer patient who came to the oncologic unit for treatment and follow-up during the data collection period and met the inclusion criteria. The first study participant was selected by using the lottery method and then data was collected from every second patient, starting with the first study participant selected randomly and continuing until the desired sample size was obtained.

### Study variables

Dependent variable: psychosocial distress.

### Independent variables

Socio demographic factors: age, sex, marital status, religion, residence, educational status, occupation, and health insurance.
Symptom burden variables: pain, fatigue(tiredness), nausea, disturbed sleep, upset, shortness of breath, problems of remembering things, lack of appetite, drowsy(sleepy), dry mouth, sadness, vomiting, and numbness.
Clinical and treatment factors: cancers type, cancer stages, cancer treatment, duration of time since diagnosis, Co-morbid disease, and performance status.
Psychosocial related factors: social support.

### Operational definitions

#### Psychosocial distress

Psychosocial distress refers to a specific co-morbid and clinically significant condition that is experienced by cancer patients. It is identified and measured by using the Questionnaire on Distress in Cancer Patients (QSC-R10), which is a validated measurement scale with psychometric properties specific to cancer. An individual with a score of >14 is recognized as having psychosocial distress, and a score of ≤14 is recognized as not experiencing psychosocial distress (44).

#### Co-morbid disease

The “coexistence of non-communicable and co-infectious diseases in addition to a primary disease of interest (cancer disease) like diabetes mellitus, hypertension, heart failure, HIV/AIDS…” (45, 46).

#### Symptoms burden

Severity of symptoms experienced by cancer patients measured by using M. D. Anderson Symptom Inventory measurement tool. Each symptom lists considered as severe if the score rated as greater or equal to 7 from a scale from 0 to 10, in which 0 (no symptom) and 10 (as bad as you can imagine) (47).

#### Perceived social support

How cancer patients perceive friends, family members, and others as sources available to provide material, psychosocial, and overall support during times of need. Measured by using Oslo three-item social support scale and considered as poor, moderate, and strong social support if scores become 3–8, 9–11, and 12–14, respectively (48).

#### Performance status

It describes a patient’s level of functioning in terms of their ability to care for themselves. Eastern Cooperative Oncology Group performance status (ECOG PS) measurement scale is used which has six grades ranging from 0 to 5. Patients with grades of ECOG PS of 0 and 1 labeled as having good performance status whereas grades of ECOG PS 2, 3, and 4 is considered as having poor performance status (49).

### Data collection tools and procedure

A structured written questionnaire was used to collect the data. It is consisted of sociodemographic factors of the respondents; clinical and treatment factors, QSC-R10; the core symptom burden list experienced by cancer patients; perceived social support; and Eastern Cooperative Oncology Group Performance Status (ECOG PS). The level of psychosocial distress was measured by using the Amharic version of QSC-R10. The QSC-R10 is a 10-item psycho-oncological screening instrument for self-assessment of psychosocial distress specific to cancer disease and its treatment. It comprises the following items: fear of disease progression, feeling tired and weak, reduced work and recreation activities, feeling tense and/or nervous, disturbed sleep, feeling physically imperfect, pain, missing partner’s empathy, few opportunities to speak with psycho-oncological professionals, and not feeling well informed about disease/treatment. Patients indicate whether each item applies to them and, if so, how severely. Thus, each item is rated on a 6-point scale ranging from 0 (the problem does not apply to me) to 5 (the problem applies to me and is a very serious problem). A total distress score is calculated by adding each item ratings. This questionnaire shows good psychometric properties, from the previous study Cronbach’s alpha for the total score is 0.85. A validated cutoff score > 14 was used to identify patients with clinical significant level of psychosocial distress, possibly requiring psychosocial-oncological treatment (44). The content validity index and the Cronbach’s alpha for this tool in this study were 0.91 and 0.89, respectively.

The core symptom burden experienced by cancer patients was assessed by using the Amharic version of the MD Anderson Symptom Inventory (MDASI) tool. The Amharic version of MDASI is a valid and reliable multi-symptom assessment tool developed for use in cancer patients. It contains 13-core symptom items rated at their severest level in the past 24h, including pain, fatigue, nausea, disturbed sleep, feeling upset, shortness of breath, difficulty remembering, lack of appetite, drowsiness, dry mouth, sadness, vomiting, and numbness or tingling. Each symptom is rated on an 11-point scale ranging from 0 to 10, with 0 = no presence of the symptom and 10 = the symptom at its highest severity level.

A symptom was severe if it was rated ≥7 on a scale of 0–10. In addition, the cut of score is reliable with alpha coefficient of 0.83 (47).

To measure level of social support among patients with cancer, the valid and reliable tool such as Amharic version of Oslo 3-item Social Support Scale (OSS-3) was used. The three items cover different fields of social support, and the OSS-3 sum score ranges from 3 to 14 and is calculated by summarizing the raw scores of the items. Scores 3–8, 9–11, and 12–14 indicate poor, moderate, and strong social support, respectively (48, 50).

To measure cancer patients’ level of performance status, the ECOG PS was used. The ECOG PS is a simple tool that is accepted and recognized by World Health Organization (WHO) and used by nurses and physicians in everyday practice to assess the functional status of patients, with a range from 0 (fully active) to 5 (dead) (49).

The Amharic versions of a written questionnaire were used to collect the data for the current research. After obtaining permission from the appropriate authorities, the data were collected by four Bachelors of science in nursing (BSC) nurses and four supervisors with BSC in nursing chosen by the researchers. All the necessary data from the sample was collected by using face-to-face interviews using structured questionnaires. Information regarding some variables, which are not known by the respondents, like cancer stage, was collected from their follow-up chart on the oncology unit.

### Data management and analysis

First data were checked for completeness and, then, it was coded and entered into Epi Data software version 3.1 and then exported to Stata statistical software version 15 for final analysis. Before analysis, missing values were checked. The outcome variable was identified as a categorical variable of psychosocial distress “caseness,” where scores >14 were coded as 1 (distressed) and scores ≤14 were coded as 0 (not distressed). Basic descriptive and summary statistics was used to describe results and computed by using frequencies, percentages, means, and standard deviation. Also, the findings were described in the form of tables, and graphs as appropriate. Binary logistic regression was used to determine statistical association between the independent and the dependent variables at 95% confidence level. All variables associated with the dependent variable with *p* < 0.25 in the bivariable logistic regression analyses were entered into multivariable logistic regression model in order to identify the association between the dependent and independent variables and to control for potential confounders. Multicollinearity was checked between independent variables through variance inflation factor for continuous independent variables and Spearman’s rank correlation for categorical independent variables. Model fitness was checked by using the Hosmer and Lemeshow’s goodness of fit test. The *p*-value for the test was 0.52, which was greater than 0.05, indicating that the model fitted the data. Variables with *p* < 0.05 in the multivariable logistic regression analyses were considered as statistically significant associated factors of psychosocial distress.

### Data quality assurance

For the assurance of data quality, an appropriate data collection tool was designed and evaluated by experienced researchers. Amharic versions of the structured questionnaire were used for data collection. First, the questionnaire was prepared in English and, then, it was translated to Amharic and back to English. A pretest on 5% of the total sample size was conducted at Tikur Anbassa Specialized Hospital oncology unit prior to three week before the commencement of the actual data collection. The pretest aimed to assess whether the checklist items are easily understood by the data collector and the study participants and to evaluate the appropriateness of the tool for the planned study. Careful modification of the checklist was done before the main study began to improve data quality. After pretest data collectors and supervisors were trained for a couple of days before the data collection on the roles, tasks, and how to ensure the confidentiality of the study participants. Close monitoring and evaluation were carried out by the supervisors and principal investigators. The collected data were reviewed and checked for completeness before data entry. The principal investigators recoded the collected data.

### Ethical considerations

The study was carried out after letter of approval was obtained from ethical review committee of Bahir Dar University, College of Medicine and Health Sciences with protocol internal review board’s decisions protocol number (CMHS/IRB 01–008). Written official letter was submitted to quality and research coordinator offices of each study areas and permission letter was also submitted for each respective oncologic unit coordinators. Objectives and purpose of the study were explained for the study participants and confirmation of the consent by the participant was obtained by signing on a consent form attached to the data collection tool. The privacy of the participants was kept during the interview. For confidentiality purposes, respondent’s personal identifier details were not required, and these were made known to them. Furthermore, all information generated from the participants was treated with confidentiality and only be reported as a group data summary without disclosing any potentiality of identifying information for any research participant.

## Results

### Sociodemographic characteristics

A total of 593 adult cancer patients participated in this study, yielding a response rate of 98.02%. Among those, 401(67.62%) were females. The mean age of the participants was 46.86 ± 14.5 years. In terms of marital status, more than two-thirds (70.15%) of the participants were married. Nearly three-quarters of the respondents (81.28%) were Orthodox Christianity. The majority, 422 (71.16%) were rural residents. More than half (54.13%) could not write and read. The majority, 338 (57%) of the respondents were farmers. Considering the respondent’s community-based health insurance (CBHI) utilization, more than half (55.65%) cancer patients utilized CBHI (**Table 1**).

**Table 1:** Sociodemographic characteristics of adult cancer patients at oncology units in the Amhara regional state, Ethiopia, 30 April to 22 June 2022 (*n* = 593).

Variables	Category	Frequency	Percent
Sex	Male	192	32.38
	Female	401	67.62
Age in years	18–24	39	6.58
	25–40	169	28.50
	41–59	254	42.83
	≥ 60	131	22.09
Marital status	Single	81	13.66
	Married	416	70.15
	Divorced	65	10.96
	Widowed	31	5.23
Religion	Orthodox	482	81.28
	Muslim	108	18.21
	Protestant	3	0.51
Residence	Urban	171	28.84
	Rural	422	71.16
Educational status	Unable to write and read	321	54.13
	Able to write and read	102	17.20
	Primary school level	49	8.26
	Secondary school level	56	9.44
	College and above	65	10.96
Occupation	Employed	62	10.46
	Unemployed	87	14.67
	Merchant	89	15.01
	Farmer	338	57.00
	Student	17	2.87
Health insurance	No	263	44.35
	Yes	330	55.65

### Clinical and treatment related characteristics

Among 593 participants, the leading type of cancer was breast cancer, accounting for 157 (26.48%). Regarding the length of time since the diagnosis of their cancer, more than half (52.11%) had a history of cancer diagnosis of less than 1-year duration. The majority of the participants were presented with advanced stages of cancer. Considering the current cancer treatment, around 330 (55.65%) patients received chemotherapy treatment alone. About 98 (16.53%) cancer patients had comorbid diseases. The majority (50.76%) of the patients had a good performance status (**Table 2**).

**Table 2:** Clinical- and treatment-related characteristics of adult cancer patients at oncology units, in the Amahra regional state, Ethiopia, 30 April to 22 June 2022 (*n* = 593).

Variables	Category	Frequency	Percent
Cancer type	Breast cancer	157	26.48
	Gynecological cancer	135	22.77
	GIT cancer	147	24.79
	Hematological cancer	93	15.68
	Others	61	10.29
Time since diagnosis	< 1 year	309	52.11
	≥ 1 year	284	47.89
Cancer stage	Stage I	48	8.09
	Stage II	49	8.26
	Stage III	197	33.22
	Stage IV	299	50.42
Current treatment type	No treatment received	54	9.11
	Chemotherapy	330	55.65
	Surgery	23	3.88
	Mixed	186	31.37
Comorbidity	No	495	83.47
	Yes	98	16.53
ECOG PS	Poor	292	49.24
	Good	301	50.76

### Core symptoms burden and psychosocial issues

The most frequently reported severe symptoms by the participants were fatigue, nausea, and pain, which accounted for 40.30%, 35.24%, and 29.17%, respectively. Nearly half (49.58%) of the patients had a history of strong social support (**Table 3**).

**Table 3:** Core symptom burden and psychosocial issue characteristics of adult cancer patients at oncology units in the Amhara regional state, Ethiopia, 30 April to 22 June 2022 (*n* = 593).

Variables	Category	Frequency	Percent
Severe pain	No	420	70.83
	Yes	173	29.17
Severe fatigue	No	354	59.70
	Yes	239	40.30
Severe nausea	No	384	64.76
	Yes	209	35.24
Sever disturbed sleep	No	445	75.04
	Yes	148	24.96
Severe feeling of upset	No	478	80.61
	Yes	115	19.39
Severe shortness of breath	No	551	92.92
	Yes	42	7.08
Severe problem on remembering things	No	553	93.25
	Yes	40	6.75
Severe lack of appetite	No	436	73.52
	Yes	157	26.48
Severe feeling of drowsy	No	557	93.93
	Yes	36	6.07
Severe dry mouth	No	548	92.41
	Yes	45	7.59
Severe feeling of sad	No	544	91.74
	Yes	49	8.26
Severe vomiting	No	508	85.67
	Yes	85	14.33
Severe numbness	No	549	92.58
	Yes	44	7.42
Social support	Strong	294	49.58
	Moderate	156	26.31
	Poor	143	24.11

### Prevalence of psychosocial distress

**Figure 1:**
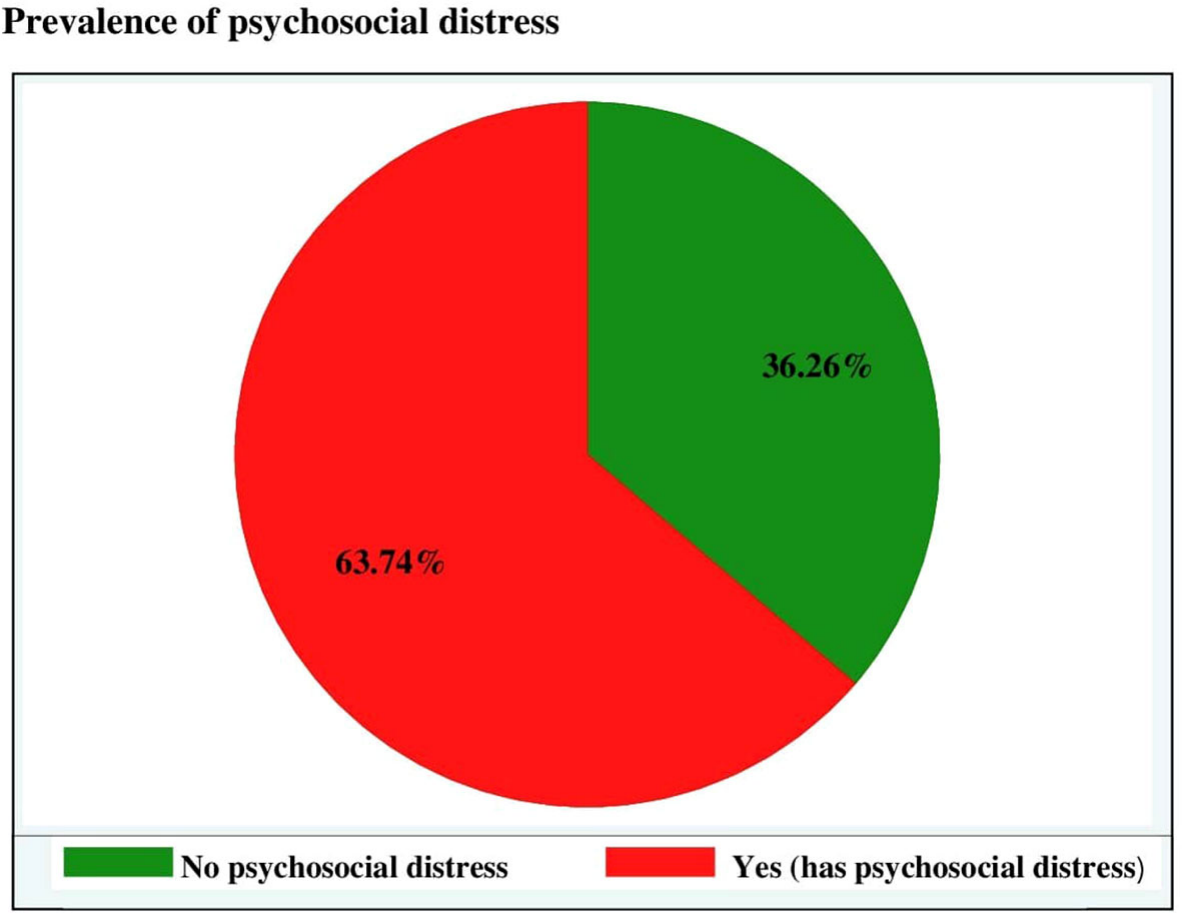
Prevalence of psychosocial distress among adult cancer patients at oncology units in the Amhara regional state, Ethiopia, 30 April to 22 June 2022.

The prevalence of psychosocial distress among adult cancer patients attending at oncology units in the Amhara regional state was found to be 63.74% (95% CI: 59.86–67.62) (**Figure 1**).

### Factors associated with psychosocial distress

A total of 28 possibly factor variables such as sex, age group, marital status, religion, residence, educational status, occupational status, status on CBHI utilization, cancer type, length of time since diagnosis of cancer, stage of cancer, treatment type, comorbidity, ECOG performance status, 13 severe symptom burdens, and level of social support to be associated with psychosocial distress were entered into the binary logistic regression model. In bivariable logistic regression, those variables such as sex, age group, residence, CBHI utilization, cancer type, cancer stage, time since diagnosis, treatment type, comorbidity, performance status, fatigue, nausea, vomiting, numbness, and level of social support were significant at 0.25 significant level. Moreover, those variables with a *p* ≤ 0.25 in the binary logistic analysis were entered into multivariable logistic analysis to identify the independent variables associated with psychosocial distress. In multivariable logistic regression analysis, seven variables such as sex, residence, health insurance, treatment type, comorbidity, fatigue, and nausea were significant at a *p* < 0.05 and 95% CI.

In this study, 272 (67.83%) female and 106 (55.21%) male adult cancer patients experienced psychosocial distress. As a result, female cancer patients were nearly twice more likely experiencing psychosocial distress when compared with male (AOR = 1.98, 95% CI: 1.24–3.17).

The likelihood of experiencing psychosocial distress was 2.3 times higher among patients living in rural area (AOR = 2.3, 95% CI: 1.49 –3.54) as compared to those who were living in urban. The odds of developing psychosocial distress were decreased by 66% among patients who utilized CBHI (AOR = 0.34, 95% CI: 0.23–0.51) as compared to patients who did not utilized community-based health insurance.

Regarding cancer treatment type, those patients who were on chemotherapy treatment were 2.72 times more likely to develop psychosocial distress as compared to those who did not start cancer treatment after adjusting other variables in the model (AOR = 2.72, 95% CI: 1.38–5.39). Cancer patients who had comorbidity disease were 3.2 times more likely to experience psychosocial distress when compared to patients who had no comorbidity (AOR = 3.2, 95% CI: 1.67–6.10). In this study, 174 (73%) adult cancer patients with severe fatigue developed psychosocial distress, whereas 204 (58.3%) patients without severe fatigue also developed psychosocial distress. In this case, the odds of experiencing psychosocial distress among severely fatigued cancer patients were increased by 1.7 times as compared to patients with no severe fatigue (AOR = 1.65, 95% CI: 1.09–2.39). The level of psychosocial distress among cancer patients with severe nausea was 155 (74.2%), whereas 223 (58.1%) of patients with no severe nausea experienced psychosocial distress. Given that the likelihood of developing psychosocial distress in cancer patients with severe nausea was almost 2 times higher than in cancer patients without severe nausea (AOR = 1.76, 95% CI: 1.10–2.80) (**Table 4**).

**Table 4:** Bivariable and multivarible logistic regression analysis of factors assocated with psychosocial distress among adult cancer patients at oncollogy units in the Amhara regional state, Ethiopia, 30 April to 22 June 2022.

Variables	Category	Psychosocialdistress		COR(95% CI)	AOR(95% CI)	*P*-value
		Yes	No			
Sex	Male	106	86	1	1	
	Female	272	129	1.71(1.20–2.44)	1.98(1.24–3.17)	**0.004**
Age group	18–24	26	13	1	1	
	25–40	95	74	0.64(0.31–1.33)	0.67(0.30–1.56)	0.354
	41–59	168	86	0.98(0.48–1.99)	1.06(0.47–2.39)	0.892
	≥ 60	89	42	1.06(0.49–2.26)	1.03(0.43–2.44)	0.953
Residence	Urban	92	79	1	1	
	Rural	286	136	1.81(1.26–2.60)	2.30(1.49–3.54)	**< 0.001**
Health insurance	Yes	185	145	0.46(0.33–0.67)	0.34(0.23–0.51)	**< 0.001**
	No	193	70	1	1	
Cancer type	Breast cancer	98	59	1	1	
	Gynecological	90	45	1.20(0.74–1.95)	0.87(0.48–1.54)	0.612
	GIT	88	59	0.90(0.57–1.42)	1.14(0.64–2.02)	0.654
	Hematological	65	28	1.40(0.81–2.45)	1.84(0.93–3.62)	0.080
	Others	37	24	0.93(0.51–1.70)	0.81(0.38–1.70)	0.578
Time since diagnosis	< 1 year	190	119	1	1	
	≥ 1 year	188	96	1.23(0.88–1.77)	1.07(0.72–1.59)	0.741
Cancer stage	Stage I	28	20	1	1	
	Stage II	26	23	0.81(0.36–1.80)	0.67(0.26–1.54)	0.396
	Stage III	123	74	1.20(0.62–2.26)	0.80(0.35–1.67)	0.499
	Stage IV	201	98	1.47(0.79–2.73)	0.90(0.41–1.98)	0.789
Current treatment type	No treatment received	24	30	1	1	
	Chemotherapy	234	96	3.05(1.69–5.48)	2.72(1.38–5.39)	**0.004**
	Surgery	12	11	1.36(0.51–3.63)	1.04(0.32–3.40)	0.944
	Mixed	108	78	1.73(0.94–3.19)	1.64(0.77–3.51)	0.202
Comorbidity	Yes	84	14	4.10(2.27–7.43)	3.20(1.67–6.10)	**< 0.001**
	No	294	201	1	1	
ECOG PS	Poor	194	98	1	1	
	Good	184	117	0.79(0.60–1.11)	0.92(0.62–1.36)	0.672
Severe fatigue	Yes	174	65	1.97(1.38–2.81)	1.65(1.09–2.50)	**0.018**
	No	204	150	1	1	
Severe nausea	Yes	155	54	2.07(1.43–3.00)	1.76(1.10–2.80)	**0.019**
	No	223	161	1	1	
Severe vomiting	Yes	59	26	1.34(0.82–2.21)	0.87(0.47–1.62)	0.664
	No	319	189	1		
Severe numbness	Yes	34	10	2.03(0.98–4.19)	1.41(0.61–3.21)	0.420
	No	344	205	1	1	
Social support	Strong	190	104	1	1	
	Moderate	92	64	0.79(0.53–1.17)	0.80(0.50–1.28)	0.358
	Poor	96	47	1.12(0.73–1.71)	1.19(0.72–1.94)	0.498

^*^P < 0.05.

Variables that are statistically significant at *P*-value <0.05 and 95% confidence interval.

## Discussion

This study identified the prevalence of psychosocial distress and factors associated with it among adult cancer patients at oncology units in the Amhara regional state, Ethiopia. From the study results, the overall prevalence of psychosocial distress among adult cancer patients was 63.74%. The magnitude of psychosocial distress in this study was relatively consistent with other studies conducted among cancer patients in Doha, Qatar (62%) (51), Sri Lanka (65%) (15), Iran (67.7%) (21). This similarity could be explained by the course of physiological (biological) similarities between cancers and their impact on psychosocial and social aspects across the countries. Whereas, this figure was to some extent higher when compared with the other previous study findings in Egypt (46%) (52), Saudi Arabia (46%) (14), South Korea where the magnitude ranged from 28.8% to 33.6% (53), across 55 North American cancer centers (46%) (12), single study in the USA (55%) (22), and multicenter study in Italy (26.6%) (13). This discrepancy could be attributed from the difference of study populations in terms of cancer types, the sociodemographic variation, study design and setting, time frame, health care inequities, and the level of country development. However, the prevalence of this study was less than studies conducted in Kenya (72.2%) (17), Cameroon (69.2%) (16), Republic of China (83.4%) (54), and Germany (89.3%) (55). The reason for this could be the studies difference in consideration of severity of psychosocial distress and the tool used for screening and diagnosis of distress with different cut of points. It is noted that these prior studies used different measurement tools other than QSC-R10. For instance, the Brief Symptom Inventory (the BSI-18), which is considered as a more conservative tool, and the NCCN distress thermometer (DT), which has been condemned as providing more false positives and the accuracy in detecting psychosocial distress is limited (56). In this study, being a female cancer patient was statistically associated with psychosocial distress, in which the likelihood of developing psychosocial distress among female cancer patients was significantly higher when compared to male cancer patients. This finding was similar to studies conducted in two sites in Japan (57, 58), Germany (59), Iran (21), and China (60). A possible reason for this finding could be that receiving a cancer diagnosis and its treatment can cause a psychosocial difficulty that can lead to cognitive and emotional crises and persistent distress among the patients (4). With regard to females, the difficulty may be more problematic for several reasons. First of all, cancer can pose more obstacles for the roles and responsibilities that females carry out than the ones that males do. Females are more likely to be confronted by ongoing household involvements due to family obligations than their male counterparts. Traditionally, females are usually regarded as caring, nurturing, compassionate, and socialized; they prioritize their families’ needs to nurture and care for others before their own needs and accomplishments. Another reason could be when women with various responsibilities manage their illnesses and the physical symptoms of the diseases at the same time, their resources could likely run out, which could ultimately cause distress. Third, females usually tend to be preoccupied with cosmetic issues, which can without any question impact their mental state and personal satisfaction. Because cancer and its treatments has cosmetic issues (58).

This study also revealed that cancer patients living in rural areas have a higher likelihood of developing psychosocial distress when compared with those living in urban areas. This finding is consistent with prior research from Iran (21) and Greece (61). This might be a result of cancer patients living in rural areas having a low perception of lifestyle modification, relaxation, and recreation, and lack of access of other luxury enjoyments in order to cope with the disease and treatment burden and lack of access or low-seeking behavior to get psychosocial and mental health counseling or consultation. Rural cancer patients face many challenges in receiving care, including problems with public transportation, distance to treatment areas, financial issues, and the availability of support when they need it (62).

According to this study, the likelihood of developing psychosocial distress among CBHI utilizers decreased by 66% when compared with cancer patients who did not utilize it. This finding was in line with research conducted in Kenya (17) and the USA (63). This could be due to the fact that CBHI provides financial protection against the cost of illness and increase access to needed health care in order to improve access to quality health services for low-income and rural households who are in need. Evidence suggested that cancer patients who do not have a medical insurance are more distressed because of the high costs of cancer therapy. At times, they sell their property, including land, to pay for the therapy (17).

Receiving chemotherapy treatment alone was associated with higher odds of developing psychosocial distress when compared to cancer patients who did not start cancer treatment. This finding was consistent with other research carried out in Kenya (17) and Brazil (64). This could be due to the fact that even if chemotherapy is one of the treatment modalities for cancer, it can bring troublesome side effects that can impact on daily living activities and can affect the emotional well-being of patients. Side effects, such as fatigue, loss of hair, together with skin color changes and loss of weight experienced by patients, lead to a disturbed body image and low self-esteem, which could lead to distress (65). Chemotherapy forces the patient to adhere to schedules of medical appointments or hospitalizations and to reallocate their roles and responsibilities, because the patient usually cannot meet obligations because of fatigue or other side effects. Another reason can be related to emotional chemo-brain, which is emotional disturbances and self-disgust resulting from the adverse drug effects of chemotherapy on the neurobiological level. Chemo-brain is thought to be a factor in the exacerbation of mental, social, and behavioral problems in physically ill people (66).

Cancer patients with comorbidities in this study were more likely to experience psychosocial distress as compared to cancer patients without comorbidities. This finding is equivalent to findings from South Korea (67) and Taiwan (45). This could be due to the fact that comorbidities having a big impact on cancer treatment decisions and management. Comorbidities may influence the progression, stage at diagnosis, treatment, and/or prognosis of cancer patients. It can limit access to the most effective and curative treatments, delay their beginning, or reduce adherence, increase toxicities of cancer treatment, and regarded as double burden for cancer patients associated with poor quality of life. Also comorbidities resulted higher health care costs (46, 68).

In this study, severe fatigue was associated with an increased likelihood of experiencing psychosocial distress. Similarly previous studies conducted in Iran (21), South Korea (69), and Vietnam (70) also identified that fatigue was a distressing symptom for cancer. This can be because severe fatigue can make performing daily routine activities more difficult, as well as making it more difficult to participate in social activities and deteriorating cognitive functions such as concentrating and remembering things. Fatigue can also decrease motivation, interest and increase frustration, irritability, and feelings of sadness.

The results of this study also showed that the likelihood of developing psychosocial distress was higher among cancer patients with severe nausea when compared to patients with no severe nausea. This result was consistent with findings from other studies (71). This could be due to the fact that nausea is still a serious side effect of cancer therapy, frequently resulting in dramatic changes in the patient’s emotions, social activities, and work situations. Patients suffering from cancer who develop nausea symptoms find it difficult to socialize and prefer to live alone rather than disrupting others’ time and feeling like a burden on their family (72).

### Limitation of the study

Since the study was a cross-sectional study, the temporal relationship between cause and effect could not be ascertained.

Second, some measurement tools used were self-rating questionnaires. This may have led to some bias.

In this study, spiritual- and income-related variables that might be considered as associated factors of psychosocial distress were not included. The Amharic version of the MDASI, a valid and reliable multi-symptom assessment tool developed for use in cancer patients that considers only severe core symptoms, was evaluated to identify whether cancer patients with severe symptom burdens had psychosocial distress. As a result, it is difficult to distinguish between mild and moderate levels of the core symptoms. This might understate the level of the potential distressing symptoms.

## Conclusion

In general, the findings of this study showed a relatively high magnitude in which around two- thirds of patients experienced psychosocial distress. The most prevalent cancer type among the participants was breast cancer. Most cancer patients were attending with advanced stages of cancer. Fatigue, nausea, and pain were the most common presenting severe symptoms experienced by cancer patients. These symptoms necessitate the prevention, early detection, and evidenced-based management by the frontline health care workers. Being female cancer patient, rural residents, CBHI utilization, chemotherapy treatment, the presence of comorbidity, and symptom severity such as severe fatigue and nausea had statistically significant association with psychosocial distress. CBHI utilization decreases the likely hood of developing psychosocial distress.

## Data availability statement

The original contributions presented in the study are included in the article/supplementary material. Further inquiries can be directed to the corresponding author.

## Ethics statement

Ethical clearance was obtained from Bahir Dar University, College of Medicine and Health Sciences, Ethical Clearance Review Committee. Managers at the hospitals provided written authorization letters. The study’s objective was explained to participants, and their written informed consent was obtained. By omitting direct personal identifiers from the questionnaire, employing code numbers, locking data with a password, and not misusing or leaking their information, confidentiality was maintained. Additionally, participants were made aware that their participation was voluntary and that they could terminate the study at any time if they felt uncomfortable about it. The issue of confidentiality and privacy was scrupulously upheld. Every technique was used in compliance with the rules and regulations that were applicable.

## Author contributions

AB: contributed to the creation of the study’s research question and design, helped write the proposal, took part in data gathering, evaluated and interpreted the findings, and wrote the manuscript. AT and SB approved the designed project with a few adjustments. They contributed to the supervision and training of the data collectors; took part in the data analysis and wrote the manuscript. The work of this project was also critically evaluated by MM, HW, MT, and MK, who also contributed to its design, participated in the analysis and interpretation of the findings, and wrote the manuscript. The compilation, review, and approval of the final manuscript were done by all authors. All authors contributed to the article and approved the submitted version.
